# Better Mental Health and Sleep under Behavioral Restrictions Due to COVID-19 in Japanese University Students: A Cross-Sectional Survey

**DOI:** 10.3390/clockssleep5030028

**Published:** 2023-07-05

**Authors:** Hideki Shimamoto, Masataka Suwa, Hiroyoshi Adachi, Manabu Adachi, Koh Mizuno

**Affiliations:** 1 Center for Education in Liberal Arts and Sciences, Osaka University, Toyonaka 560-0043, Japan; 2 Department of Food and Nutrition, Koriyama Women’s University, Koriyama 963-8503, Japan; suwa@koriyama-kgc.ac.jp; 3 Campus Life Health Support/Consultation Center, Osaka University, Toyonaka 560-0043, Japan; hadachi@psy.med.osaka-u.ac.jp; 4 Faculty of Human Health, Sonoda Women’s University, Amagasaki 661-8520, Japan; m-adachi@sonoda-u.ac.jp; 5 Faculty of Education, Tohoku Fukushi University, Sendai 981-8522, Japan; mizuno-k@tfu.ac.jp

**Keywords:** COVID-19, mental health, sleep, Japanese university students

## Abstract

Coronavirus disease (COVID-19) is a global pandemic, which is not only a severe public health issue but also significantly impacts the physical activity, sleep habits, and mental health of university students. Thus, we examined the association between behavioral restrictions due to COVID-19 and sleep patterns and mental health in first-year Japanese university students. Four hundred and twenty-two students (253 males and 169 females; age, 18.7 ± 1.0 years) participated in our questionnaire study. Under the behavioral restrictions due to COVID-19, 193 students (127 males and 66 females) responded to the questionnaire online from home. The participants did not visit the university during the survey period. The data acquired the year before the COVID-19 pandemic (2018 and 2019) were used as control data (126 males and 103 females). The questionnaire consisted of four sections: (1) demographic and lifestyle variables, (2) the Pittsburgh Sleep Quality Index, (3) the Japanese version of the Epworth Sleepiness Scale, and (4) the Patient Health Questionnaire-9. Our data revealed that self-restraint due to COVID-19 was associated with better sleep and mental health. In addition, mental health was independent of sleep, while sleep was related to mental health. These differences were more pronounced in male than in female students. This finding could be due to physical activity at night, part-time work, and long commuting times during the pre-pandemic period.

## 1. Introduction

Coronavirus disease (COVID-19) was first reported in December 2019 and was determined to be caused by a novel coronavirus in January 2020. On 11 March 2020, the World Health Organization declared COVID-19 a global pandemic. This infectious disease is a severe public health issue that has resulted in significant health, educational, and social problems.

On 7 April 2020, Japan declared its first state of emergency. A lockdown could not be enforced because of the legal system; however, significant behavioral restrictions were observed. Similar to other countries, the lifestyle of Japanese people changed significantly. Governments requested strict home confinement measures, with a few exceptions. Schools, universities, and shops selling non-essential goods were temporarily suspended. In 2020, almost all undergraduate university courses from spring to summer transitioned to an online mode.

Even without an epidemic, students entering university undergo significant lifestyle changes, including changes in their sleep and physical activity. The starting time for university varies from person to person. Many students live alone for the first time and begin part-time jobs. They also participate in extracurricular activities. These major lifestyle changes may lead to psychological and physical stress in university students. Buchanan et al. mentioned that university age is one of the most stressful factors in a person’s life [1]. Thus, mental health issues are more prevalent in university students than in other students.

The sleep problems of university students are often associated with mental health issues. Students with insomnia commonly experience mental health issues, such as chronic fatigue, depression, stress, decreased optimism, anxiety, and diminished quality of life [2]. A significant percentage of university students worldwide fulfill the diagnostic criteria for insomnia, ranging from 9.4% to 13.1% [3]. Sleep disorders, poor sleep quality, and excessive daytime sleepiness are associated with lower academic motivation and self-efficacy [4]. For these reasons, the relationship between mental health and sleep should be considered in depth in this age group.

Many previous studies have reported changes in sleep patterns and mental health of university students in a COVID-19 environment. It has been reported that the spread of coronavirus infection has deteriorated the quality of sleep. Marelli et al. [5] found a delay in bedtime, sleep latency, and wake-up time before and during the COVID-19 emergency, as well as worsening sleep quality and insomnia symptoms in university students. Cellini et al. [6] reported that sleep–wake rhythms markedly changed, with people going to bed and waking up later, spending more time in bed, and reporting lower sleep quality. Similarly, previous studies have reported that mental health was also significantly worse during COVID-19 emergencies [7,8].

The sleep situation in Japan impacted the results of this study significantly. Steptoe et al. [9] reported that Japanese university students have the shortest sleep duration and poorest self-rated health worldwide. Morita et al. [10] reported that 11% of Japanese university students were diagnosed with insufficient sleep syndrome, a clinical condition characterized by daytime sleepiness, shorter sleep duration on weekdays, and longer sleep duration on weekends or holidays. This study demonstrated that participants with insufficient sleep syndrome had worsened mental health status. As for the sleep–wake pattern, living conditions (alone/with family) and daily activities were associated with a late bedtime [11,12].

Therefore, it is meaningful to examine the association between behavioral restrictions due to COVID-19 and sleep habits and mental health in Japanese university students. It is also interesting to consider the effect on sleep–wake rhythms in Japanese people who originally had a short sleep time. Here, we examined the association between behavioral restrictions due to COVID-19, and sleep patterns and mental health in first-year Japanese university students.

## 2. Results

### 2.1. Characteristics of Participants and the Results of the Questionnaire Survey

Table 1 shows the characteristics of the participants and the results of the questionnaire survey before and during the COVID-19 outbreak. The percentage of students with exercise habits was significantly lower during the COVID-19 pandemic than before COVID-19. The commuting time of 55.0 min became 0 min because the students could not attend university during COVID-19. The results of Patient Health Questionnaire-9 (PHQ-9), Epworth Sleepiness Scale (ESS), and Pittsburgh Sleep Quality Index (PSQI) were significantly lower during COVID-19, but the percentage above the cut-off value was significant only in ESS before COVID-19. During COVID-19, bedtime and wake-up time were significantly later, and sleep time and latency were significantly longer.

The characteristics of the participants and the results of the questionnaire survey by gender are also shown in Table 1. The percentage of students with exercise habits was significantly lower during COVID-19 than before COVID-19 in both groups. The commuting time was 55.5 min for males and 54.8 min for females before COVID-19, but it was 0 min during COVID-19 because students could not attend university. The PHQ-9 and global PSQI scores were lower during the COVID-19 pandemic but were statistically significant only for males. The ESS score was significantly lower during COVID-19 for both males and females, and the percentage above the cut-off value was also significant for both sexes. Bedtime was delayed during COVID-19 but was statistically significant only in males. Sleep latency was longer during the COVID-19 pandemic in both males and females. During COVID-19, wake-up time and bedtime were statistically significant for both sexes.

### 2.2. Association of Self-Restraint Due to COVID-19 with Global PSQI Score

The associations between self-restraint due to COVID-19 and the global PSQI scores using multivariate linear regression models are shown in Table 2 and Table 3. In all participants, the global PSQI score was inversely associated with self-restraint due to COVID-19 (β = −0.142, *p* = 0.004), gender, age, and BMI-adjusted (β = −0.131, *p* = 0.009) models, and gender, age, BMI, exercise habits, and living situation-adjusted (β = −0.123, *p* = 0.016) models. In males, the global PSQI score was inversely associated with self-restraint due to COVID-19 (β = −0.145, *p* = 0.021), age, and BMI-adjusted (β = −0.138, *p* = 0.032) models, and age, BMI, exercise habits, and living situation-adjusted (β = −0.134, *p* = 0.044) models. In females, there was no significant association between self-restraint due to COVID-19 and global PSQI scores. In all participants, the associations of self-restraint due to COVID-19 with the global PSQI score were eliminated by adjusting the PHQ-9 score in model 4. In other words, although behavioral restrictions due to COVID-19 seemed to relate to sleep quality and patterns, the relation would be mediated by mental health.

### 2.3. Association of Self-Restraint Due to COVID-19 with PHQ-9 Score

The associations of self-restraint due to COVID-19 with the PHQ-9 score using multivariate linear regression models are shown in Table 4 and Table 5. In all participants, the PHQ-9 score was inversely associated with self-restraint due to COVID-19 in all models. In model 4, there was a significant inverse association between self-restraint due to COVID-19 and the PHQ-9 score after adjusting for the global PSQI score (β = −0.124, *p* = 0.012). This result indicates that improvements in mental health during COVID-19 were independent of sleep. The PHQ-9 score was inversely associated with self-restraint due to COVID-19 in all models in males, whereas the PHQ-9 score was unrelated to self-restraint due to COVID-19 in females.

## 3. Discussion

Our data show that sleep and mental health in Japanese university students are better under conditions of behavioral restrictions due to the COVID-19 pandemic. This study observed that students had longer sleep duration, improved sleep quality, less daytime sleepiness, and improved mental health under home confinement compared to a regular lifestyle. Interestingly, this result differs from those of many previous studies that reported poor sleep and mental health during the COVID-19 pandemic. In addition, our results revealed that the improvement in mental health was independent of sleep and that sleep was better because of the improvement in mental health. When examined by gender, these alterations were more pronounced in the male participants.

Even under normal conditions, sleep problems are known to be closely related to mental health issues in university students. Sleep problems affect the physical and mental health of university students and their daytime functioning. In fact, previous studies suggested that many university students have poor sleep quality [13], which has been linked to lower academic performance in university students without depression [4]. It is common for students with insomnia to experience mental health problems such as chronic fatigue, depression, stress, lower optimism, anxiety, and a lower quality of life. Sleep patterns change considerably from high school to university because of alterations in external time triggers such as class schedules and lifestyle preferences [14]. Academic demands, part-time jobs, friends, family, relationships, lectures, and free-time activities may contribute to a delay in sleep [13]. These lifestyle habits, especially postponement of sleep, are suspected to induce irregular sleep patterns [15]. Short sleep duration is a risk factor for poor perceived health and persistent psychological distress [16,17]. Difficulties in falling asleep and staying asleep are common complaints among university students [3,18]. Thus, poor sleep quality and deterioration of mental health caused by these factors can also lead to the deterioration of academic performance.

Behavioral restrictions due to the COVID-19 outbreak have exposed most individuals to an unprecedented stressful situation of unknown duration. This may not only increase daytime stress, anxiety, and depression levels but also disrupt sleep [19]. Being forced to stay at home, work from home, drastically minimize outings, reduce social interaction or work many more hours under stressful circumstances, and, in parallel, manage the attendant health risks can significantly impact daily functioning and nighttime sleep [19]. During the COVID-19 pandemic, three studies examined sleep and psychological distress among Japanese university students. A longitudinal study that compared sleep habits before and after the COVID-19 pandemic (from 2018 to 2020) showed increased sleep duration on weekdays, possibly due to remote teaching [20]. Another study that evaluated sleep problems by examining digital media use suggested that students whose screen time increased during 2019 and 2020 were at higher risk for sleep problems [21]. Finally, Iio et al. [22] surveyed Japanese first-year university students in mid-April 2022 and found that 27.4% of the participants experienced psychological distress due to sleep deprivation. Although our study was a cross-sectional survey conducted before and after the COVID-19 pandemic, the results of longer sleep duration after the COVID-19 pandemic were comparable to those reported by Hori et al. Although the questionnaires used to evaluate psychological distress were different, the percentage of students suffering from psychological stress was higher (27.4%) in the study reported by Iio et al. than that in the present study (11.4%). Because the years of data correction were 2022 [22] and 2020 (the present study), the continuation of behavioral restrictions due to the COVID-19 pandemic resulted in a higher percentage of students with psychological stress.

As expected, many previous studies have shown that sleep quality and duration deteriorate during the self-restraint period owing to COVID-19. Several studies have evaluated the differences in sleep before and during COVID-19 assessed through sleep duration, bedtime/wake time, and sleep quality. Changes in bedtime/wake time are rather consistent, suggesting both delayed bedtime and delayed wake time during COVID-19 [6,23]. Some studies have reported slight increases in sleep duration [23,24]. Studies examining differences in sleep quality have shown poorer sleep quality during the COVID-19 pandemic. In Italy, Casagrande et al. found that 57% of their sample reported poor sleep quality during the lockdown, a percentage considerably higher than that reported for Italians in separate pre-pandemic studies [25]. Similarly, in two Italian studies [5,6] and studies from Germany, Switzerland, and Austria [24], sleep quality was shown to be poorer during lockdown relative to pre-lockdown. Gao and Scullin found that sleep quality was significantly worse during the lockdown [26]. Considering these results, self-restraint due to COVID-19 seems to have a negative impact.

However, our data clearly showed longer sleep duration, improved sleep quality, less daytime sleepiness, and improved mental health during home confinement due to COVID-19. Few studies have reported similar results. Benham [27] reported that stress, sleep quality, and insomnia were not significantly higher in the samples collected during the COVID-19 pandemic, bedtime and wake time were significantly later during the pandemic, and sleep duration was significantly longer. His results suggest that, within the US college student population, the impact of COVID-19 on stress and sleep may not be entirely negative. Gao and Shullin [26] demonstrated that the average sleep quality was unchanged or even improved early in the pandemic. However, there were clear individual differences. Approximately 25% of participants reported that their sleep quality had worsened, which was explained by stress vulnerability, caregiving, adverse life impacts, work shift, and COVID-19 symptoms. Gao and Shullin [26] indicated that the negative impact of morning commitments, the urgency of work/school demands, and extensive to-do lists can affect sleep. In the case of university students, the results of these previous studies might have been affected by factors such as morning commitments and school demands.

In this study, self-restraint due to COVID-19 was associated with better sleep quality, and this association was mediated through mental health. Previous studies have also reviewed a causal relationship in which a lower state of mental health negatively affects sleep [28,29]. The findings of this study are consistent with those studies.

The university entrance examination system in Japan is rather strict [30,31]. It is easy to guess that a long commuting time is stressful for university students immediately after entering the university. Lifestyle changes—such as part-time jobs, extracurricular activities, late activity hours, and diversity of class hours—also had negative effects on both mental and physical activities. In addition, it can be said that reduced stress by eliminating the commuting time and on-campus studying was more prevalent than the deterioration of sleep quality due to the night type. In addition, the fact that Japanese people originally had short sleep durations and could sleep freely through university closures may have impacted the results of this study. Previous studies have reported that Japanese sleep time is the shortest worldwide [9]. The OECD published similar results [32]. Steptoe et al. [9] also noted that Japanese university students with shorter sleep times had poor self-rated health. Further, it has been pointed out that long commuting times are stressful for young people [33]. These aspects of the Japanese lifestyle may have affected the results of this study, and further studies are needed to confirm this hypothesis on a global scale.

Our data indicated that when examined by gender, alterations in mental health and sleep were more pronounced in male participants. There is a widely known difference in the risk of sleep problems and depression between males and females [34]. Previous studies have reported that sleep and mental health declines are more pronounced in females [35], who are two times more likely to experience depression [36], but total sleep and slow-wave sleep durations were longer in females [9,37]. In other words, there is some disagreement in the data, and it can be said that various factors have a complex influence on each other.

Few studies have compared the mental health of students according to gender during the COVID-19 pandemic. Essadek and Rabeyron [38] reported that females scored significantly higher on mental health measures than did males in France. On the other hand, Cao et al. [39] found no significant difference between genders when studying college students in China. Furthermore, Meda et al. [40] explored the mental health of university students before, during, and after the COVID-19 lockdown in Italy and reported that students experienced worse depressive symptoms during the lockdown, independent of gender. These results seem to vary depending on pandemic time and restrictions. Thus, gender differences in mental health and sleep in the COVID-19 pandemic environment remain unknown. Further research is required to address this issue.

Our study had some limitations. First, because of the unexpected and extraordinary circumstances of the COVID-19 pandemic, it was not possible to obtain longitudinal data starting before the COVID-19 pandemic. Second, because this was a cross-sectional survey, a causal relationship could not be determined. Third, physiological measurements could not be used to obtain objective data for the evaluation of sleep habits. In practice, it is difficult to obtain detailed physiological data using an epidemiological study design. A previous study has shown that self-reported sleep data are correlated with physiological data [41]. Fourth, in the present study, questions concerning underlying sleep disorders were not posed in the questionnaire. As insomnia symptoms may be caused by underlying sleep disorders, questions concerning such disorders must be included in the questionnaires for future studies. Items that were not included in this study must be examined in the future. Fifth, the difficulty in getting a part-time job during the pandemic affected students’ mental health. However, the lack of need for additional income from a part-time job may be associated with improved sleep quality and mental health. Additionally, similar dietary habits should be considered, and these factors should be investigated in future studies.

Our data indicate that sleep and mental health in Japanese university students are better under behavioral restriction conditions due to the COVID-19 pandemic. Under these conditions, mental health is in a better state, independent of sleep, while sleep is in a better state because of better mental health. Physical activity at night, part-time work, and long commuting times in the normal environment might have influenced this result in our experimental group.

## 4. Materials and Methods

### 4.1. Participants

The questionnaire survey of this study was conducted in May and June 2020. This period was the first self-restraint period due to COVID-19. The first state of emergency in Japan was declared on 7 April 2020. The Japanese government notified each municipality of appropriate behavioral restrictions in schools and universities. In response to this notice, the surveyed universities decided to prohibit students from entering after April 14. University courses were taught remotely via the internet, and students took online courses from their homes. All extracurricular activities were prohibited. When they responded to the questionnaire, students were not attending university.

Four hundred and twenty-two first-year university students (253 males and 169 females; age, 18.7 ± 1.0 years) participated in this study. Under the behavioral restrictions due to COVID-19, 193 students (127 males and 66 females) responded to the online questionnaire from home using the university network. Owing to the pandemic, the students did not attend university during the survey period.

The data acquired the years before the COVID-19 pandemic (2018 and 2019) were used as control data (126 males and 103 females). These control data were obtained from May to June, corresponding to the period of this study during the COVID-19 pandemic. In the control group, face-to-face surveys were conducted among the participants.

Excluding incomplete answers, the response rate was 87.9% before COVID-19 and 88.9% during COVID-19.

### 4.2. Ethics Approval

The ethics committee of Osaka University approved the study (approval code E20-131016). After being fully informed of the nature of the study protocol, which involved surveying the lifestyle and health status of university students, the participants provided informed written consent and were allowed to participate in the study.

### 4.3. Questionnaires

The questionnaire consisted of the following four sections: (1) demographic and lifestyle variables, (2) Pittsburgh Sleep Quality Index (PSQI), (3) Epworth Sleepiness Scale (ESS), and (4) Patient Health Questionnaire-9 (PHQ-9).

#### 4.3.1. Demographic and Lifestyle Variables

The demographic and lifestyle variables of the participants were examined using a self-administered questionnaire designed by the authors. The question items included name, age, gender, undergraduate course, extracurricular exercises, and part-time jobs.

#### 4.3.2. PSQI

The second section contained the PSQI, which was used to measure sleep quality and patterns over the last month [42,43,44]. The PSQI is the gold standard questionnaire for assessing subjective sleep quality and has been validated in both clinical and non-clinical populations [42,45]. The questions were rated on a 4-point Likert scale (0–3) and included seven factors, including subjective sleep quality, sleep latency, sleep duration, habitual sleep efficiency, sleep disturbances, use of sleeping medications, and daytime dysfunction. The scores from each component were added to give a sum score, also called the global score (range, 0–21) [45]. As in the original version, a score of ≥6 indicated sleep disturbance. The cut-off score was 5.5 points.

#### 4.3.3. ESS

The ESS was used to measure daytime sleepiness [46,47]. The questionnaire required the participant to rate eight different situations on a scale from 0 to 3. The higher the score, the greater the sleepiness level. The cutoff value was 11, and participants with a score >11 were deemed to have excessive daytime sleepiness.

#### 4.3.4. PHQ-9

The PHQ-9, a scale known as a major depressive disorder module, was used to assess depressive tendencies. This questionnaire was used to diagnose depression and grade the severity of symptoms in general medical and mental health settings [48,49]. Scores of each of the nine Diagnostic and Statistical Manual of Mental Disorders criteria of major depressive disorder were ranked from “0” (not at all) to “3” (nearly every day), providing a 0–27 severity score. Higher PHQ-9 scores indicated greater depressive tendencies and depression severity. A cutoff score ≥10 was used as the threshold for depression.

### 4.4. Statistical Analysis

Data are shown as mean ± SD.

In the comparison between before and during COVID-19, the percentages of students with exercise habits and the percentage of those living alone were compared using the χ2 test. In addition, the ratios above the cutoff values of the PHQ9, ESS, and PSQI were also tested using the χ2 test. For other variables, the Mann–Whitney U test was used.

Multiple linear regression models were used to estimate the association between self-restraint due to COVID-19 and the PSQI (Table 2 and Table 3). Before and during COVID-19 (before: 0; during: 1) was used as the explanatory variable. In the analysis of all participants, the following four models were used to assess these associations: Model 1 was crude; model 2 was adjusted for gender, age, and BMI; model 3 was adjusted for model 2 covariates plus exercise habits and living situation; and model 4 was adjusted for model 3 covariates plus PHQ-9 score. In the analysis by gender, the following four models were used to assess these associations: Model 1 was crude, model 2 was adjusted for age and BMI, model 3 was adjusted for model 2 covariates plus exercise habits and living situation, and model 4 was adjusted for model 3 covariates plus PHQ-9 score.

Similarly, these models were used to estimate the association between self-restraint due to COVID-19 and the PHQ-9 (Table 4 and Table 5). Before and during COVID-19 (before: 0; during: 1) was used as the explanatory variable. In the analysis of all participants, the following four models were used to assess these associations: Model 1 was crude; model 2 was adjusted for gender, age, and BMI; model 3 was adjusted for model 2 covariates plus exercise habits and living situation; and model 4 was adjusted for model 3 covariates plus global PSQI score. In the analysis by gender, the following four models were used to assess these associations: Model 1 was crude, model 2 was adjusted for age and BMI, model 3 was adjusted for model 2 covariates plus exercise habits and living situation, and model 4 was adjusted for model 3 covariates plus global PSQI score.

All calculated variance inflation factors were <1.14.

All statistical analyses were performed using SPSS version 25.0 (IBM, Armonk, NY, USA). Differences were considered statistically significant at *p* < 0.05.

## Figures and Tables

**Table 1:** Characteristics of the participants and the results of the questionnaire survey before and during the COVID-19 outbreak.

		All			Male			Female		
		before COVID-19	during COVID-19	*p*-Value *	before COVID-19	during COVID-19	*p*-Value *	before COVID-19	during COVID-19	*p*-Value *
Number of participants		229	193		126	127		103	66	
Age	yr	18.9 (1.1)	18.5 (0.8)	<0.001	19.0 (0.9)	18.6 (0.8)	<0.001	18.9 (1.3)	18.4 (0.6)	<0.001
Stature	cm	165.4 (9.2)	168.2 (9.1)	0.003	171.6 (6.5)	173.4 (6.0)	0.042	157.8 (5.8)	158.2 (4.7)	0.854
Body weight	kg	56.7 (10.2)	58.2 (9.8)	0.028	62.0 (10.5)	62.9 (7.8)	0.168	50.3 (4.8)	49.3 (6.6)	0.148
BMI	m^2^/kg	20.7 (2.8)	20.5 (2.4)	0.901	21.1 (3.4)	20.9 (2.4)	0.793	20.2 (1.9)	19.7 (2.4)	0.14
Exercise habits #	%	26.2	11.3	0.001	31.7	15.0	0.003	19.4	6.0	0.015
Commuting time	minutes	55.0 (38.3)	0 (0)	<0.001	55.5 (37.7)	0 (0)	<0.001	54.8 (39.2)	0 (0)	<0.001
Living situation #	% of living alone	32.3	38.3	0.196	30.2	41.7	0.055	35.0	31.8	0.674
PHQ-9	score	5.5 (4.0)	4.3 (3.9)	<0.001	5.6 (4.3)	4.0 (3.9)	0.001	5.5 (3.7)	4.8 (3.8)	0.153
Above cutoff of PHQ-9 score #	%	13.5	11.4	0.509	13.5	10.2	0.423	13.6	13.6	0.993
ESS	score	11.4 (4.4)	9.6 (3.8)	<0.001	11.1 (4.4)	9.4 (3.7)	0.001	11.9 (4.4)	10.1 (4.1)	0.009
Above cutoff of ESS score #	%	58.9	37.3	<0.001	56.3	34.6	0.001	62.1	42.4	0.017
Sleep Quality Index (PSQI)	score	5.6 (2.1)	4.9 (2.3)	0.003	5.6 (2.0)	5.0 (2.4)	0.015	5.4 (2.1)	4.8 (2.0)	0.083
Above cutoff of PSQI score #	%	45	40.4	0.345	44.4	40.9	0.574	45.6	39.4	0.425
Bedtime	hh:mm	0:29 (0:57)	0:42 (1:05)	0.012	0:33 (0:56)	0:47 (1:05)	0.032	0:23 (0:57)	0:32 (1:05)	0.33
Sleep latency	min.	14.9 (14.5)	24.3 (20.7)	<0.001	15.6 (14.0)	24.9 (20.4)	<0.001	14.1 (15.2)	23.1 (21.4)	<0.001
Wake-up time	hh:mm	7:15 (1:01)	8:15 (1:07)	<0.001	7:16 (0:57)	8:20 (0:59)	<0.001	7:14 (1:05)	8:05 (1:19)	<0.001
Sleep duration	hh:mm	6:15 (0:57)	7:03 (0:51)	<0.001	6:12 (0:56)	7:01 (0:52)	<0.001	6:17 (0.59)	7:06 (0:51)	<0.001

Data are mean (SD). * *p* values from the Mann–Whitney U test or χ^2^ test. # χ^2^ test.

**Table 2:** Relationship between self-restraint due to COVID-19 and the global PSQI score using multivariate linear regression models in all participants.

	Partial Regression Coefficient	SE	β	95% CI	*p*-Value
Model 1 *	−0.618	0.211	−0.142	−1.032 to −0.203	0.004
Model 2 **	−0.573	0.217	−0.131	−1.000 to −0.146	0.009
Model 3 ***	−0.537	0.223	−0.123	−0.975 to −0.100	0.016
Model 4 ****	−0.358	0.218	−0.082	−0.785 to 0.070	0.101

* Crude, R^2^ = 0.020, adjusted R^2^ = 0.018. ** Adjusted for gender, age, and BMI; R^2^ = 0.026; adjusted R^2^ = 0.017. *** Adjusted for gender, age, BMI, exercise habits, and living situation; R^2^ = 0.028; adjusted R^2^ = 0.013. **** Adjusted for gender, age, BMI, exercise habits, living situation, and PHQ-9 score; R^2^ = 0.094; adjusted R^2^ = 0.079.

**Table 3:** **Table 3.** Relationship between self-restraint due to COVID-19 and the global PSQI score using multivariate linear regression models by gender.

	Partial Regression Coefficient	SE	β	95% CI	*p*-Value
**Male**					
Model 1 *	−0.643	0.277	−0.145	−1.188 to −0.098	0.021
Model 2 **	−0.614	0.284	−0.138	−1.173 to −0.054	0.032
Model 3 ***	−0.593	0.293	−0.134	−1.170 to −0.015	0.044
Model 4 ****	−0.361	0.285	−0.081	−0.923 to 0.201	0.207
**Female**					
Model 1 *	−0.628	0.330	−0.146	−1.281 to 0.024	0.059
Model 2 **	−0.490	0.336	−0.114	−1.154 to 0.174	0.147
Model 3 ***	−0.428	0.344	−0.099	−1.107 to 0.251	0.215
Model 4 ****	−0.285	0.335	−0.066	−0.947 to 0.377	0.396

* Crude. Male: R^2^ = 0.021, adjusted R^2^ = 0.017. Female: R^2^ = 0.021, adjusted R^2^ = 0.015. ** Adjusted for age and BMI. Male: R^2^ = 0.024, adjusted R^2^ = 0.012. Female: R^2^ = 0.051, adjusted R^2^ = 0.033. *** Adjusted for age, BMI, exercise habits, and living situation. Male: R^2^ = 0.025, adjusted R^2^ = 0.005. Female: R^2^ = 0.058, adjusted R^2^ = 0.029. **** Adjusted for age, BMI, exercise habits, living situation, and PHQ-9 score. Male: R^2^ = 0.106, adjusted R^2^ = 0.084. Female: R^2^ = 0.123, adjusted R^2^ = 0.091.

**Table 4:** Relationship between self-restraint due to COVID-19 and the PHQ-9 score using multivariate linear regression models.

	Partial Regression Coefficient	SE	β	95% CI	*p*-Value
Model 1 *	−1.227	0.387	−0.153	−1.987 to −0.467	0.002
Model 2 **	−1.199	0.399	−0.150	−1.984 to −0.414	0.003
Model 3 ***	−1.255	0.408	−0.157	−2.057 to −0.453	0.002
Model 4 ****	−0.997	0.397	−0.124	−1.777 to −0.217	0.012

* Crude, R^2^ = 0.023, adjusted R^2^ = 0.021. **Adjusted for gender, age, and BMI; R^2^ = 0.026, adjusted R^2^ = 0.017. *** Adjusted for gender, age, BMI, exercise habits, and living situation; R^2^ = 0.034, adjusted R^2^ = 0.020. **** Adjusted for gender, age, BMI, exercise habits, living situation, and global PSQI score; R^2^ = 0.100, adjusted R^2^ = 0.085.

**Table 5:** Relationship between self-restraint due to COVID-19 and the PHQ-9 score using multivariate linear regression models by gender.

	Partial Regression Coefficient	SE	β	95% CI	*p*-Value
**Male**					
Model 1 *	−1.556	0.514	−0.188	−2.569 to −0.543	0.003
Model 2 **	−1.573	0.528	−0.190	−2.614 to −0.533	0.003
Model 3 ***	−1.487	0.542	−0.179	−2.555 to −0.419	0.007
Model 4 ****	−1.171	0.525	−0.141	−2.204 to −0.137	0.027
**Female**					
Model 1 *	−0.633	0.591	−0.083	−1.800 to 0.535	0.286
Model 2 **	−0.649	0.605	−0.085	−1.844 to 0.547	0.286
Model 3 ***	−0.947	0.601	−0.124	−2.134 to 0.240	0.117
Model 4 ****	−0.750	0.585	−0.098	−1.904 to 0.405	0.202

* Crude. Male: R^2^ = 0.035, adjusted R^2^ = 0.031. Female: R^2^ = 0.007, adjusted R^2^ = 0.001. ** Adjusted for age and BMI. Male: R^2^ = 0.035, adjusted R^2^ = 0.024. Female: R^2^ = 0.025, adjusted R^2^ = 0.008. *** Adjusted for age, BMI, exercise habits, and living situation. Male: R^2^ = 0.045, adjusted R^2^ = 0.026. Female: R^2^ = 0.087, adjusted R^2^ = 0.059. **** Adjusted for age, BMI, exercise habits, living situation, and global PSQI score. Male: R^2^ = 0.125, adjusted R^2^ = 0.103. Female: R^2^ = 0.150, adjusted R^2^ = 0.119.

## Data Availability

Data sharing not applicable.
